# Deficits of psychomotor and mnesic functions across aging in mouse lemur primates

**DOI:** 10.3389/fnbeh.2014.00446

**Published:** 2015-01-09

**Authors:** Solène Languille, Agatha Liévin-Bazin, Jean-Luc Picq, Caroline Louis, Sophie Dix, Jean De Barry, Olivier Blin, Jill Richardson, Régis Bordet, Esther Schenker, Fathia Djelti, Fabienne Aujard

**Affiliations:** ^1^UMR 7179, Centre National de la Recherche Scientifique, Muséum National d’Histoire Naturelle FranceBrunoy, France; ^2^Laboratoire de Psychopathologie et de Neuropsychologie, EA 2027, Université Paris 8St-Denis, France; ^3^Institut de Recherches ServierCroissy-sur-Seine, France; ^4^Eli Lilly, Erl Wood ManorSurrey, UK; ^5^INCI UPR3212 CNRS et Innovative Health DiagnosticsStrasbourg, France; ^6^Centre de Pharmacologie Clinique et d’Evaluations Thérapeutiques-CIC, Timone CNRS-INT-Aix Marseille UniversitéMarseille, France; ^7^GlaxoSmithKline, R&D China U.K. Group StevenageStevenage, UK; ^8^Département de Pharmacologie Médicale, EA 1046, Université Lille Nord de France, UDSL, Faculté de MédecineCHU, Lille, France

**Keywords:** psychomotor, anxiety, working memory, spatial memory, recognition memory

## Abstract

Owing to a similar cerebral neuro-anatomy, non-human primates are viewed as the most valid models for understanding cognitive deficits. This study evaluated psychomotor and mnesic functions of 41 young to old mouse lemurs (*Microcebus murinus*). Psychomotor capacities and anxiety-related behaviors decreased abruptly from middle to late adulthood. However, mnesic functions were not affected in the same way with increasing age. While results of the spontaneous alternation task point to a progressive and widespread age-related decline of spatial working memory, both spatial reference and novel object recognition (NOR) memory tasks did not reveal any tendency due to large inter-individual variability in the middle-aged and old animals. Indeed, some of the aged animals performed as well as younger ones, whereas some others had bad performances in the Barnes maze and in the object recognition test. Hierarchical cluster analysis revealed that declarative-like memory was strongly impaired only in 7 out of 25 middle-aged/old animals. These results suggest that this analysis allows to distinguish elder populations of good and bad performers in this non-human primate model and to closely compare this to human aging.

## Introduction

Human aging is characterized by a complex process which associates various alterations of different biological functions and more specifically a decline in psychomotor and mnesic functions (Morris and McManus, 1991). Not all individuals, and not all cognitive domains, are equally affected by cerebral aging. Whereas some older people perform as well as the young and even outperform the younger in some cases, older people generally perform worse than younger people on most of cognitive tasks. “Mild Cognitive Impairment” has an estimated prevalence of 15–20% in the elderly (Lopez et al., 2003) and has been proposed as an early phase in cognitive decline that precedes severe dementia, such as Alzheimer’s disease (Petersen et al., 2001). With the increase of the lifespan in developed countries, the study of aging needs to be addressed seriously to understand the biological bases and associated diseases, and to prevent or treat the most debilitating conditions of old age.

Animal models of aging and associated neurodegenerative diseases represent a critical step for the understanding of the mechanisms of brain aging as well as for efficient drug development. Transgenic rodent models remain the most widely used and have provided extensive insight in the field of cognitive aging, but have low predictive validity for clinical trials outcomes (Jucker, 2010). Physiological models of aging and neurodegenerative processes as non-human primate represent an interesting alternative to transgenic animals (Lavery, 2000). The gray mouse lemur (*Microcebus murinus*), a non-human primate, has attracted researcher’s attention and seems highly relevant to study aging (Languille et al., 2012b). They present a lot of advantages: (i) small size (~60–120 g), rapid maturity and high fecundity, (ii) relatively short lifespan (8–12 years), (iii) several genetic, physiological and anatomical similarities with humans, (iv) aged-related heterogeneity, like observed in the human population (Languille et al., 2012b). With regard to cerebral functions, several studies on age-related changes in mouse lemurs have demonstrated the relevance of this model for the study of normal and pathological cerebral aging. Indeed, neuritic plaque-like deposits and neurofibrillary changes in certain neurons have been reported in the brain of some aged mouse lemurs (Bons et al., 1992, 1994; Mestre-Francés et al., 1996), as well as pathological tau metabolism (Delacourte et al., 1995), neurochemical alterations (Jallageas et al., 1998), iron accumulation (Dhenain et al., 1998), neuronal loss in some specific cerebral structures such as the nucleus basalis of Meynert (Mestre and Bons, 1993) and atrophy of specific brain regions (Dhenain et al., 2000). The brain atrophy has been related to some mnesic deficits in mouse lemurs (Picq et al., 2012), reinforcing the relevance of this model to study cerebral aging. In a behavioral point of view, some psychomotor declines (Némoz-Bertholet and Aujard, 2003), working memory deficit (Picq, 1993, 1995; Trouche et al., 2010), and spatial reference memory deficit (Picq et al., 2012) have been previously described in old mouse lemurs, and point out the greater variability in older animals. However these investigations were performed separately in different groups of animals. Thus, a relationship between different behaviors has not yet been evaluated. Since psychomotor decline and other age-related behavioral changes (such as anxiety and motivation) could be connected to the mnesic performance (Granholm et al., 2008), and since individuals and specific functions are differently affected by aging, it seemed essential to perform a behavioral screening of mouse lemurs at different stages of life, i.e., young, middle-aged and old adults. By using combined analyses in the same individual, the present study on aging of psychomotor and memory in a non-human primate is the first of its kind.

The present study aimed at: (1) evaluating psychomotor capacities, anxiety-related behaviors and mnesic performances in mouse lemurs across aging using newly adapted tests; (2) defining the stage of life at which sudden or progressive changes occur; (3) identifying relationships between mnesic and non-mnesic functions, and between each mnesic function (spatial and non-spatial; short- and long-term memories); and (4) diagnosing various degrees of mnesic deficits in this species by using hierarchical cluster analysis. Forty-one mouse lemurs were evaluated in a series of potentially age-sensitive behavioral tests, adapted to mouse lemur ecology. Psychomotor capacities were tested in a hang, rotarod, and a wire/rope balance task, to assess strength, endurance, balance and coordination capacities, respectively. Anxiety-related behaviors were evaluated in an open-field and a light/dark plus-maze test. Spatial working memory was tested in a spontaneous alternation task in a cross-maze. Spatial reference and novel object recognition (NOR) memory tasks (declarative-like memory tasks; i.e., memory for facts and events) were performed both at short- (less than 1 h) and long- (24 h) term delays.

## Materials and methods

### Animals and experimental design

Forty-one gray mouse lemurs (*Microcebus murinus*) were attributed to three age categories: 13 young adults (6 females and 7 males) from 31 to 51 months old (mean = 40.3 ± 2.1 m.o.), 16 middle-aged adults (10 females and 6 males) from 59 to 81 months old (mean = 70.3 ± 1.9 m.o.), and 12 old adults (6 females and 6 males) from 84 to 113 months old (mean = 100.7 ± 2.9 m.o.). These age categories were based on survival data of the breeding colony which has a mean and maximum lifespan of 56 and 120 months, respectively (Languille et al., 2012b). There are no significant differences in body mass between these three groups. The animals were born and reared in the Brunoy colony (MNHN, France, license approval N° A91.114.1). During this study, they were housed in cages in same-sex pairs. The cages (50 × 49 × 50 cm) were equipped with wooden branches and wooden nests (nestbox) and were kept at standard temperature (24–26°C) and relative humidity (55%). In the colony, animals were kept in alternating 6-month period of long-days (14:10 light/dark, summer-like photoperiod) and short-days (10:14 light/dark, winter-like photoperiod). Light was provided by cool fluorescent lamps (250–350 lux) and a dim red light (about 0.002 lux) was provided during the dark phase. To avoid the effect of age-related hormonal fluctuation on behavior, which occurs during the long-day period (Aujard and Perret, 1998; Perret, 2005), all experiments were performed during the short-day period. The animals were provided daily (immediately before the nocturnal phase) with fresh fruits (6 g of apple and 6 g of banana) and 15 g of a mixture composed of cereal, milk and egg. Water was provided *ad libitum*. All studies were ethically reviewed and carried out in accordance with the European Communities Council Directive 86/609/EEC and the GSK Policy on the Care, Welfare and Treatment of Animals. All experiments were performed under authorization n°91–582 delivered by the “Direction Départementale de la Protection des Populations de l’Essonne”.

All behavioral tests were carried out during the last 4 h before the nocturnal phase, with an interval of at least 24 h between two consecutive tests, and in the same chronological order during 8 weeks. All of the experiments and video analyses were observer-blinded for the age category (as far as possible due to some age-related morphological differences) and the animals were evaluated in a random order each day. The eyes of the lemurs were examined by a veterinary ophthalmologist and no anomalies (e.g., cataract, sclerosis of the lens) were detected that would affect visual acuity.

### Psychomotor tasks

#### Hang task

The grip strength of animals was evaluated by their ability to hang on a border of a smooth cylinder (55 cm long, Ø 8 cm) positioned vertically, with 2 slits (9.5 cm long, 0.5 cm width) at 34 cm above the floor. The apparatus was dried between the three trials, separated by a 1-min inter-trial interval (ITI), to avoid animals to slide down the cylinder. Latency to falling off was recorded. Animals that did not fall off during the 10 min trial period were removed and the maximum score of 600 s was attributed. One young animal (51 m.o.) did not grasp the slit and jumped on the floor at each trial.

#### Accelerating rotarod task

The measurement of endurance performance was addressed by using an accelerating (from 17 to 40 rpm) motor-driven treadmill (a rotarod, Ø 5 cm, model 7750, Ugo Basile, Italy). The running time spent on the rotarod before falling or gripping on the rod during at least three consecutive turns was recorded. Animals underwent five consecutive trials (ITI = 1 min) and the best result was recorded (max 60 s). Animals that jumped consistently from the rotarod at each trial (6 young, 5 middle-aged and 1 old animals) were excluded from the analysis.

#### Balance task

The balance and the coordination of animals were tested by observing the locomotion on a wire bar (72 cm long, Ø 2 mm, with 45° slope) or on a tight rope (55 cm long, Ø 2 mm, without slope) freely suspended (81 cm from the floor) in a closed chamber (55 × 58 × 148 cm). To motivate the animal to move on the rope, its nestbox was placed at the end of the wire or the rope. For each material (wire or rope), animals had three successive trials (ITI = 1 min). All tests were video-taped, allowing for fine measures of the time to cross and the total number of loss of balance on either rope or wire before reaching the nestbox during the three trials. One young animal (31 m.o.) that jumped to the nestbox without crossing on the rope, and one old animal (99 m.o.) that did not perform with either material were excluded from the analysis.

### Anxiety-related tests

#### Open-field test

Each mouse lemur was individually placed in a corner of a square open-field (95 × 95 × 25 cm; lighted with 60 W white light in each corner) via an airlock (112 cm^2^). After a 30 s resting phase, the door was removed and the animal was allowed to explore the novel environment. The latency of the first movement (distance of at least 14 cm), and the duration of movement in the center zone (50 × 50 cm) of the open-field during the 30-min session were recorded using a video camera and Ethovision XT7 (Noldus). Before each test, the whole area was cleaned.

#### Light/dark plus-maze test

Four cylindrical arms (30-cm long, Ø 9 cm) were arranged in a cross-like formation, with two dark arms (black walls) opposite of each other and two light arms (transparent walls) that intersected at a central square platform (16.5 × 16.5 cm), which provided access to any of the four arms. All of the floor surfaces were black, and the central platform was under homogeneous illumination at 60 W (previously described in Languille et al., 2012a). Each animal was allowed to freely explore the apparatus for 5 min while its behavior was video-taped. The following variables were recorded: the latency before the first visit to the dark arms and light arms, and the time spent (with all four paws) inside each arm. If the animal did not visit the arm, a latency of 300 s was indicated. The percentage of time spent in the light arms was calculated in relation to the total time spent in both types of arms. Two young animals which did not visit the apparatus were excluded from the analyses of the percentage. Two young, 2 middle-aged and 2 old animals were not tested due to implementation of this new test during the experiment.

### Spatial working memory task: spontaneous alternation test

The test was performed in a cross-shaped maze (each arm: 40 × 15 × 25 cm, illuminated by a red 15 W light bulb). The four arms ended with 90° left turns (10 × 15 × 25 cm) which are not visible from the center of the maze. Different signs cover the internal wall of each arm. At the start of the trial, each animal was placed in the maze center, and was allowed to explore the maze during 10 min. The number and the sequence of visits (all four paws into one arm) were recorded. The animal must show a tendency to enter a less recently visited arm (using working memory). Alternation was defined as successive entries into the four arms on overlapping quadruplet sets in which four different arms are entered. The possible alternation sequences are equal to the number of arm entries minus 3. The percentage of alternation is equal to the ratio of (actual alternation/possible alternation) × 100. Only data from animals that made at least 10 arm entries (i.e., seven possible alternations of 4 arms) were included in the statistical analyses (5/13 young, 8/16 middle-aged and 7/12 old animals).

### Spatial reference memory task: barnes maze test

The apparatus consisted of a circular platform separated into 12 compartments located at the periphery (detailed description in Languille et al., 2012a). Each compartment contained one circular hole giving access to a black Plexiglas nestbox, which served as refuge. Fourteen objects (with different forms, sizes and colors) were attached around the periphery of the platform to serve as extra-maze visual cues. Before each trial, the platform and the target box were cleaned, and the platform was randomly rotated on its central axis to avoid the use of intra-maze cues. For each trial, the mouse lemur was individually placed in the center of the platform. If the animal did not explore the maze after 2 min, the maze was gently shaken. The animal had to reach the target box positioned beneath one of the 12 holes, which was kept constant relative to the visual cues in all trials. When the animal entered the target compartment, the target box was opened to allow the animal to escape from the maze and the trial was stopped; the animal remained in the target box for 2 min.

On the first day, each animal was given two 5-min trials of learning in a four-walled chamber containing only the open target compartment (one-choice test), which provided access to the target box. The short-term (3 min after the last learning trial) and long-term (24 h later) memories were evaluated by a 10-min trial involving 12 open compartments, but with only the target compartment providing access to the target box. Performance was assessed by the number of errors (e.g., the number of entries into compartments containing the non-escape holes).

### Novel object recognition (NOR) task

NOR memory test, adapted from rodent studies, was fitted for the first time to mouse lemurs with the following procedure. NOR tests were performed in a T-maze and used three different ITIs (5 min, 1 h and 24 h). The tests were repeated twice for each ITI, thus all animals participated in six NOR tests, using six different sets of objects. The order of the ITIs was randomized for each animal.

#### Apparatus

The start arm (42 × 13 × 15.5 cm) of the T-maze was separated from the goal arms (25 × 15 × 15.5 cm each) by a sliding door, allowing that animals went quietly into the goal arms. Both goal arms can give access to a nestbox through the opening of the door at the left- and the right- end of the maze. The experimenter was separated visually from the animal by a screen with a one-way mirror. Trials occurred in darkness except for a red light attached to the front of the T-maze that gave a bright illumination without shadow and assured that the animal could not see the experimenter. The three arms and the nestboxes were wiped down between each trial.

#### Objects

We used six different sets of plastic objects that varied in shape, color, and size (volume between 6.3 and 24.6 cm^3^). The objects were fixed in place with Patafix® to withstand the investigative behavior of mouse lemur. The objects were meticulously washed after each use with a 2% solution of household bleach to eliminate any possible odor cues. Moreover, there were three copies of each object, so none of the two objects from the sample trial had to be used as the familiar object in the choice trial. Our preliminary studies have shown that a delay of 2 days minimum and a modification of the locations of the objects-sets were required between NOR tests to improve objects exploration in mouse lemur.

#### Habituation procedure

Before the first NOR test, a three-phase habituation procedure was used to prepare the animal for exploration. Each mouse lemur was individually and progressively habituated to the T-maze in 1 day, i.e., animal was allowed to explore the apparatus (without any objects) six times for 10 min maximum (ITI = 3 min). Firstly, the animal was habituated to visit separately the right arm and the left arm during two trials: only one goal arm was opened and gave access to a nestbox when the animal was close to the nestbox door. Secondly, the animal was habituated to visit the right and the left arms during two trials: both goal arms were opened and the animal had access to the nestbox when both arms were visited. Thirdly, the animal was habituated to visit twice both arms during two trials: both goal arms were opened and animal had access to the nestbox when both arms were visited twice.

#### Tests procedure

Each NOR test included three trials of 10-min max each. During the T0 trial, animal was habituated to the T-maze as in the last trials of the habituation day. During the sample trial (T1), duplicate copies of an object (A1 and A2) were placed in a symmetrical position, with one object on each goal arm of the T-maze. The animal was then allowed to explore during 10 min max. After the sample trial, delays were imposed before exposure to the T-maze in the choice trial. For the 5 min and 1 h ITIs, the animal was placed in its nestbox with the hole oriented to the floor of a cage. This position prevented the animal from being exposed to external visual stimuli. For the 24 h ITI, the animal was returned to its cage in the colony room. The animal was always maintained alone in its nestbox, and 4 to 6 animals were placed in covered cage for all transportations between the colony room and the experimental apparatus. Subsequently, after a ITI, the animal was put back in the apparatus for the choice trial (T2) during 10 min, but now with two different objects: a familiar one (A3, the third copy of the triplicate set of the objects used in the sample trial) and a new one (B).

#### Data treatment

Each trial was recorded by an infra-red video camera located above the apparatus, which allowed off-line analysis of the exploratory behavior of the mouse lemur. The time spent exploring each object during T1 and T2, and the latency of the first exploratory behavior were recorded manually by an observer blind for animal age, for the ITI and for object novelty. Indeed, the amount of time spent exploring objects during the choice trial was scored before measuring the amount of exploration during the sample trial. This reverse-order of analysis ensured that the scorer was blind with respect to which object was familiar and which object was new. Object location and objects (familiar and novel) were used in a balanced manner among the age categories to reduce potential bias of location or object preference. An animal was considered exploring when its nose was directed towards an object at a distance of maximally 2 cm or when touching the object with its nose. Climbing on an object, sitting on it or resting against it was not considered an exploratory behavior. Animals which did not explore any of the objects during T1 and/or T2 were excluded from the analysis. The difference in time spent exploring the novel object compared with the familiar object divided by the time spent exploring both objects was calculated to obtain the “discrimination ratio” (the highest value of each ITI was analyzed). One animal of each age category had no discrimination ratio at 24 h ITI.

### Statistical analysis

For all statistical assessments, data were first tested for normality using R 2.12.1 software. A Kruskal-Wallis test (*χ*^2^ value) was performed, followed by a *post-hoc* analysis using a Mann-Whitney test to compare age categories. Since there is a greater variability in the middle-aged and old groups when compared to young animals, linear regressions were also performed for each parameter by using Kendall-theil robust line-fit method. The Fisher’s exact test was used to compare the proportion of animals of each group which expressed some specific behaviors (e.g., moved in center zone of the openfield, loss of balance on the support). The Wilcoxon Signed-Rank test was used to compare the short-term vs. the long-term memory performances, and also the exploration time of the familiar object vs. the exploration time of the novel object in NOR task. To display the mnesic performance relationships between animals as a dendrogram, hierarchical cluster analysis was performed using the Ward’s minimum variance method and by treating the observations of the 37 mouse lemurs (some values of Barnes or NOR parameters were lacking for 1 young, 2 middle-aged and 1 old animals) as Euclidean distances in an agglomerative fashion. The height from one animal to another along the path of the dendrogram is related to the similarity of their patterns of declarative-like memory (Barnes and NOR tests). A *p*-value of <0.05 was considered significant. All values are given as the median and interquartile (IQ: lower quartile-upper quartile) in the text and are represented by scatter plots with median in the figures.

## Results

### Psychomotor functions

**Figure 1:**
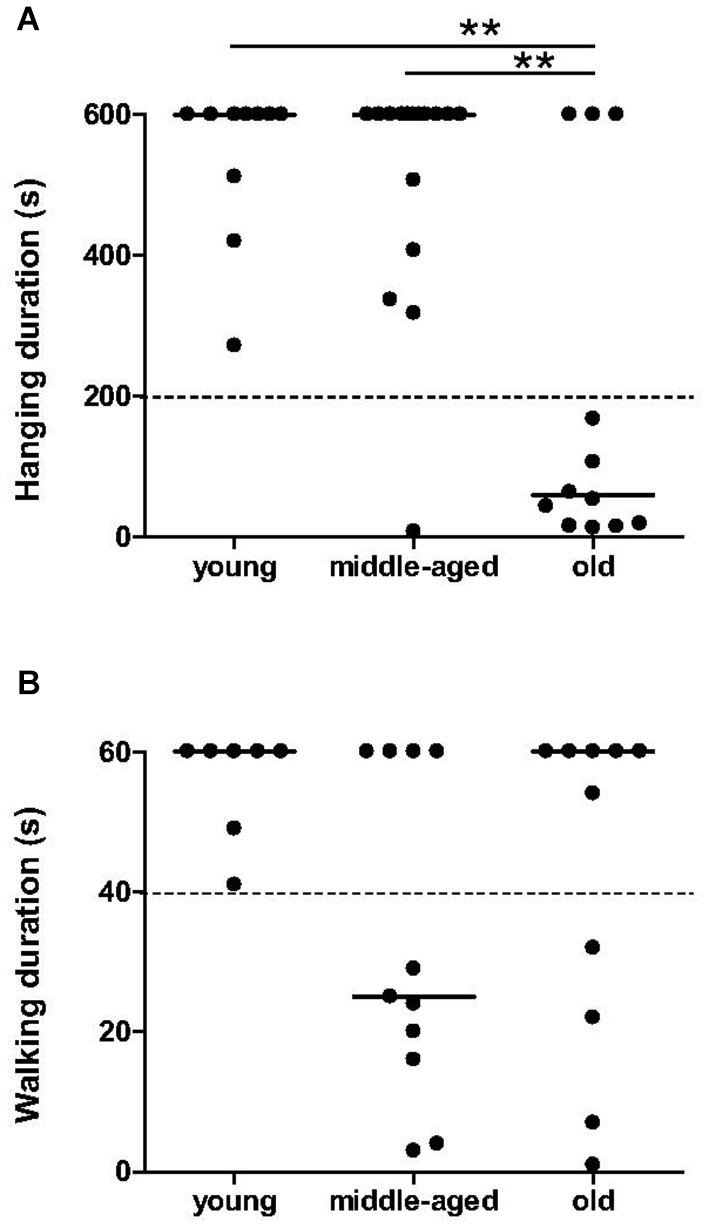
**Muscle capacities as function of age categories at the hang task and in accelerating rotarod task**. Muscle strength in hang task was affected in almost all old mouse lemurs **(A)**; hanging duration inferior to 200 s was indicated by dotted line. Endurance on rotarod was less than 40 s (indicated by dotted line) only in some middle-aged and old animals **(B)**. Medians are represented by solid line. **p < 0.01.

Significant effect of age was observed in the hang task (*χ*^2^ = 12.08, *p* = 0.0024, Figure 1A). The duration of hanging on the cylinder was significantly shorter for old animals compared to young (*p* = 0.0023) and middle-aged animals (*p* = 0.0059). Most of the young and middle-aged mouse lemurs were able to hang at least for 200 s, while the majority of the old animals (9 among 12) fell off within 200 s. Therefore, grip strength of mouse lemurs seems to decrease suddenly when animals are older than 84 months.

The walking time on the rotarod did not differ significantly between the age categories (*χ*^2^ = 4.24, *p* = 0.12, Figure 1B). However, a tendency towards a decrease in endurance of older animals was observed: 7 among 11 middle-aged and 4 among 11 old animals spent less than 40 s on the rotarod, while all young animals remained over 40 s. Young animals present as an uniform group for endurance capabilities, whereas middle-aged and old animals seem to divide into two distinct groups with good or bad endurance.

**Figure 2:**
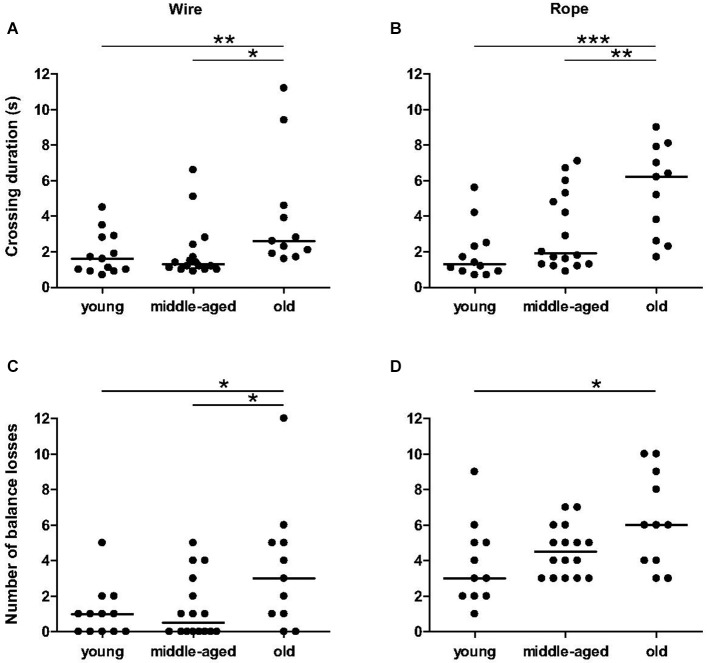
**Balance and coordination capacities as function of age categories in balance task**. The duration of crossing of old animals was significantly higher than young and middle-aged animals on wire **(A)** and rope **(B)**. The number of balance losses was different between young and old animals for wire **(C)** and rope **(D)**. Medians are represented by solid line. ***p < 0.001, **p < 0.01, *p < 0.05.

Agility performances on the wire bar and rope became significantly worse with aging. Independent on the material used, there are significant differences between age categories for duration of the crossing (wire: *χ*^2^ = 8.37, *p* = 0.015, Figure 2A; rope: *χ*^2^ = 13.014, *p* = 0.0015, Figure 2B) and total number of balance losses (wire: *χ*^2^ = 5.86, *p* = 0.050, Figure 2C; rope: *χ*^2^ = 6.17, *p* = 0.046, Figure 2D). Especially, in any of the two settings, old animals crossed more slowly than young (*p* = 0.026 for the wire, Figure 2A; *p* = 0.0009 for the rope, Figure 2B) and middle-aged animals (*p* = 0.005 for the wire, Figure 2A; *p* = 0.016 for the rope, Figure 2B). Old animals exhibited more loss of balance on the wire bar than young (*p* = 0.043, Figure 2C) and middle-aged animals (*p* = 0.042, Figure 2C). On the rope, the number of balance losses of old animals was only significantly different than those from young animals (*p* < 0.05, Figure 2D). No significant differences were observed between young and middle-aged animals for all parameters. These data indicate that balance and coordination seem to be impaired only in old mouse lemurs.

### Anxiety-related behaviors

**Figure 3:**
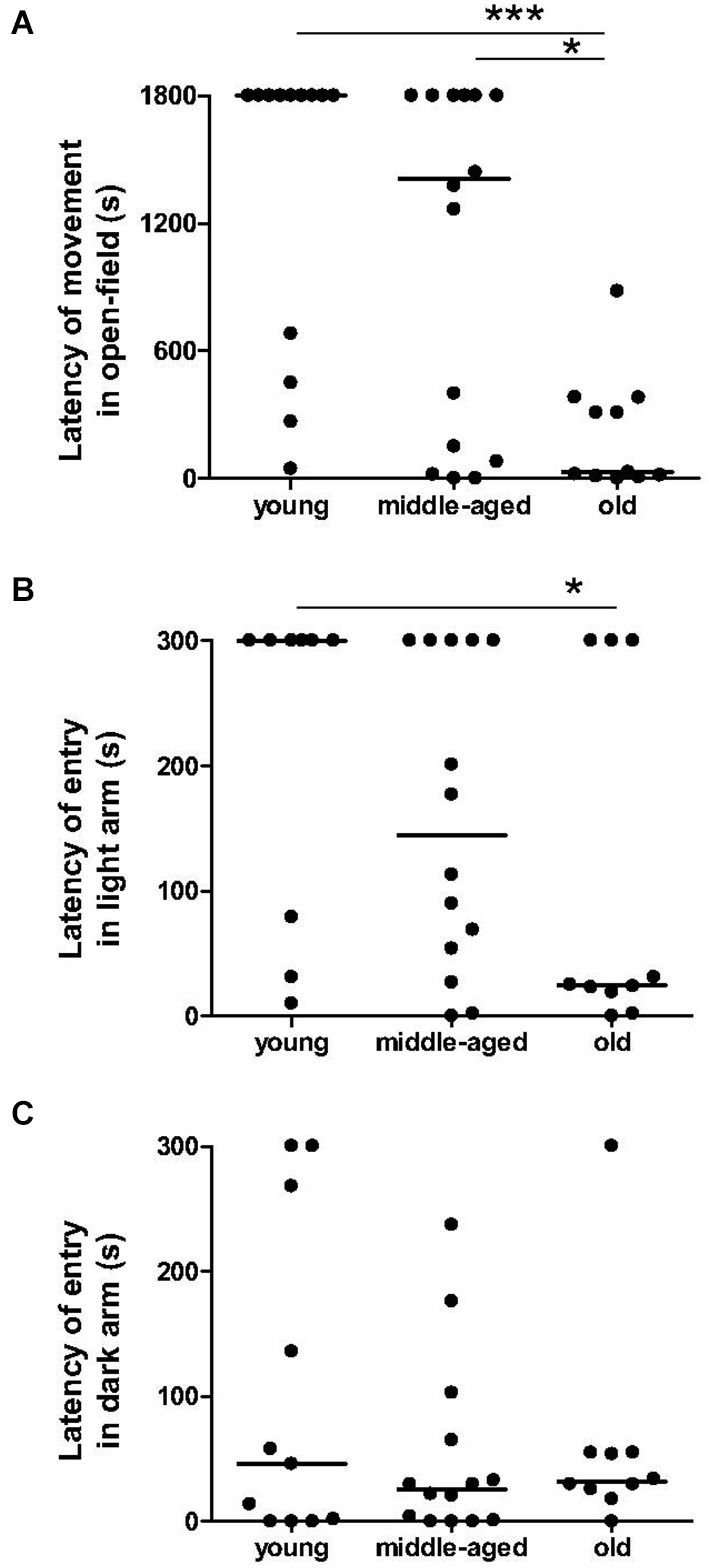
**Anxiety-related behavior as function of age categories in the open-field test and in the light/dark plus-maze test**. In open-field **(A)**, the latency of first movement of old animals is lower than the young and middle-aged animals. Moreover, old animals took less time to start exploration of a lighted arm **(B)**, but not of an unlighted arm **(C)** than young animals. Medians are represented by solid line. ***p < 0.001, *p < 0.05.

The number of animals that moved in the center zone did not differ significantly between age categories (3 among 13 young, 7 among 16 middle-aged, and 6 among 12 old animals; *p* > 0.1), and the time spent in center zone did not differ with age: 0 s (IQ: 0 s–0.55 s) for young animals, 0 s (IQ: 0 s–1.35 s) for middle-aged animals and 0.3 s (IQ: 0–6.3 s) for old animals (*p* > 0.2). However, the latency to the first movement in the open-field differed significantly with age (*χ*^2^ = 13.3, *p* = 0.0013, Figure 3A). Old animals started exploration in the open-field much earlier than middle-aged (*p* = 0.014) and young (*p* = 0.0004) animals. No significant difference was observed between the latter two groups (*p* = 0.17).

Percentage of time spent in the dark arms of the light/dark plus-maze did not differ between age categories: 100% (IQ: 56.5%–100%) for young animals, 92% (IQ: 48.5%–100%) for middle-aged animals and 78.5% (IQ: 54%–100%) for old animals (*p* > 0.4). Again, latency of the first entry into the dark arm was constant across age (*χ*^2^ = 0.66, *p* = 0.72, Figure 3C), whereas the latency of the first entry into the light arm was significantly higher in young than in old animals (*χ*^2^ = 4.70, *p* = 0.03, Figure 3B). In this way, latency of first movement in the open-field was positively correlated with latency of first entry into the light arm (*r*^2^ = 0.26, *p* = 0.002, Table 2), but not with latency of entry into the dark arm (*r*^2^ = 0.001, *p* = 0.84).

**Table 1:** **Exploration time of the familiar and novel object in novel object recognition task in function of the inter-trial interval (ITI) and the age categories**.

ITI	Age categories	Exploration time (sec)		*p* value
		Familiar object	Novel object
5-min	Young	0.0 (0–0.32)	2.4 (0.62–4.61)	0.00016
	Middle-aged	0.1 (0–0.45)	1.9 (1.02–5.32)	0.000038
	Old	0.4 (0–0.92)	3.4 (1.16–4.5)	0.000098
1-h	Young	0.4 (0–1.13)	3.2 (1.31–6.16)	0.00066
	Middle-aged	0.2 (0–0.66)	2.0 (0.55–6.3)	0.000018
	Old	0.3 (0–1.39)	1.8 (0.50–3.43)	0.019
24-h	Young	0.0 (0–0.88)	1.6 (0.75–2.94)	0.00085
	Middle-aged	0.3 (0–1.03)	2.4 (0.78–3.47)	0.00057
	Old	0.4 (0.22–1.13)	1.9 (0.78–3.53)	0.044

*Values are expressed as the median and interquartile in bracket (lower quartile-upper quartile). All the p-values of the paired test (comparison of the exploration time of the familiar object vs. novel object) are less than the 0.05 significance level*.

**Table 2:** **Spearman’s correlation coefficients matrix (*r* squared values) for all potential relationships between the psychomotor function, anxiety-related behavior and mnesic performance**.

		Psychomotor function					Anxiety-related function			Mnesic function					
		Walking duration	Crossing duration on wire	Crossing duration on rope	Balance losses on wire	Balance losses on rope	Latency in openfield	Latency in light arm	Latency in dark	Spontaneous alternation	Errors at 3 min in Barnes maze	Errors at 24 h in Barnes maze	NOR discrimination at 5 min	NOR discrimination at 1 h	NOR discrimination at 24 h
**Psychomotor function**	Hanging duration	0.00	**0.44** ***p* < 0.001**	0.13	**0.26** ***p* < 0.01**	0.00	0.08	0.00	0.04	0.18	0.00	0.00	0.02	0.11	0.04
	Walking duration		0.01	0.04	0.04	**0.31** ***p* < 0.01**	0.00	**0.46** ***p* < 0.01**	0.17	0.11	0.01	0.05	0.10	0.01	0.02
	Crossing duration on wire			**0.53** ***p* < 0.001**	**0.52** ***p* < 0.001**	0.00	**0.23** ***p* < 0.05**	**0.15** ***p* < 0.05**	0.00	0.03	0.01	0.07	0.01	0.01	0.04
	Crossing duration on rope				**0.44** ***p* < 0.001**	0.06	**0.24** ***p* < 0.05**	**0.29** ***p* < 0.01**	0.02	0.01	0.03	0.07	0.01	0.00	0.00
	Balance losses on wire					0.02	0.12	0.09	0.00	0.09	0.02	0.01	0.02	0.03	0.01
	Balance losses on rope						0.01	**0.37** ***p* < 0.001**	0.00	0.11	0.05	0.01	0.01	0.01	0.07
**Anxiety-related function**	Latency in openfield							**0.26** ***p* < 0.01**	0.00	0.02	0.02	0.06	0.03	0.11	0.03
	Latency in light arm								0.02	0.11	0.01	0.02	0.00	0.00	0.07
	Latency in dark arm									0.17	0.02	0.00	0.00	0.01	0.03
**Mnesic function**	Spontaneous alternation										0.02	0.00	0.04	0.07	0.02
	Errors at 3 min in Barnes maze											**0.18** ***p* < 0.05**	0.00	0.03	0.01
	errors at 24 h in Barnes maze												0.01	0.10	0.00
	NOR Discrimination at 5 min													0.00	0.04
	NOR Discrimination at 1 h														0.02

*Only significant p-values (p < 0.05, p < 0.01 or p < 0.001) are expressed in the table. Whereas some psychomotor parameters are correlated amongst themselves and with some anxiety-related parameters, mnesic parameters are not correlated with psychomotor or anxiety-related parameters, and amongst themselves (except errors at 3 min and at 24 h in Barnes maze)*.

### Spatial working memory tasks: spontaneous alternation test

**Figure 4:**
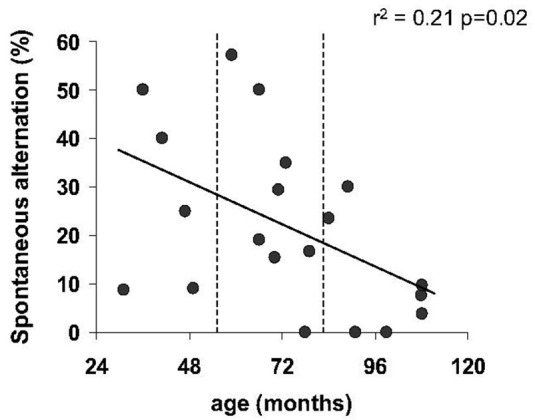
**Spatial working memory as function of age in spontaneous alternation task**. Regression line showed a significant decline of percentage of spontaneous alternation with age.

The number of visits in the cross-maze did not differ significantly with age (*p* = 0.19). Moreover, Spearman’s correlation revealed no relationship between the number of arms visited and the percentage of alternation (*r*^2^ = 0.0009, *p* = 0.89). Linear regression identified a negative relationship between the percentage of spontaneous alternation and age (*r*^2^ = 0.21, *p* = 0.025, Figure 4), revealing a significant decreasing of spontaneous alternation with age. It should be noted that two young animals (31 and 49 m.o.) showed a low percentage of alternation.

### Spatial reference memory task: barnes maze test

**Figure 5:**
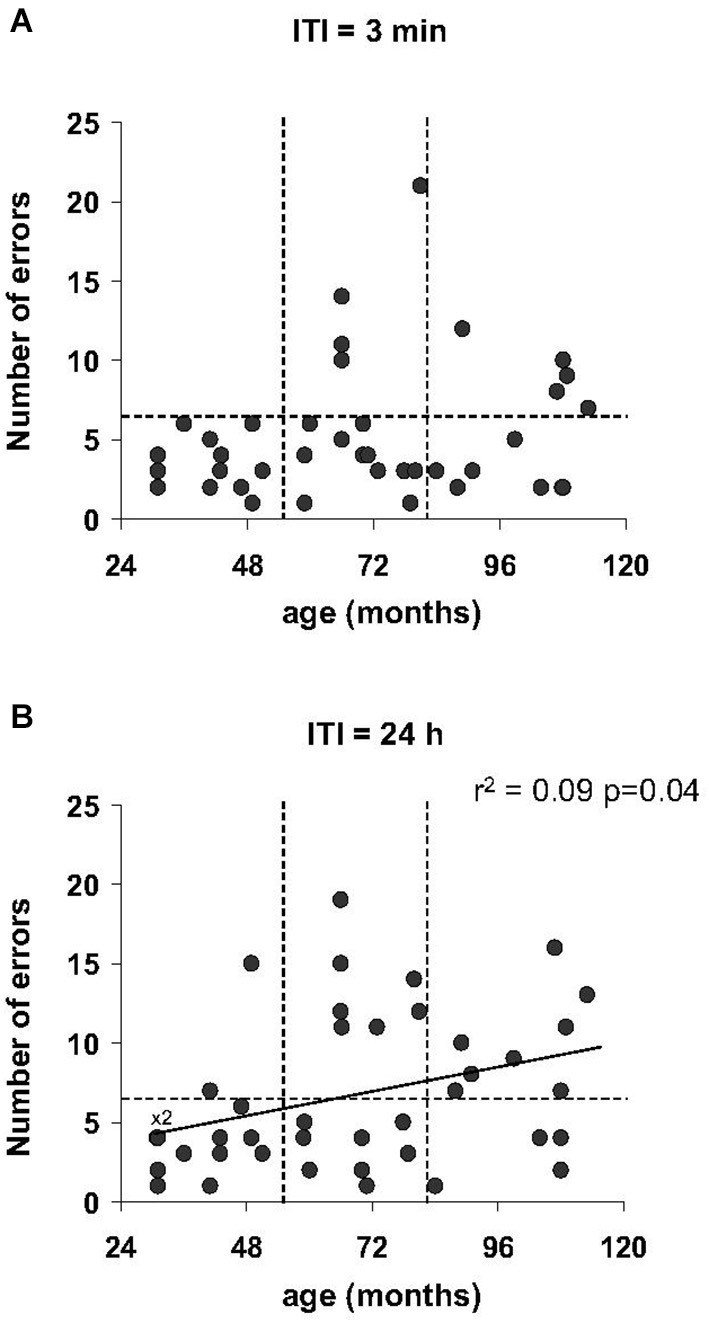
**Spatial reference memory as function of age in the Barnes maze test**. The number of errors prior to reaching the target tends to increase with age at 3 min ITI **(A)** which became significant at 24 h ITI **(B)** with linear regression. More than 5 errors are indicated by dotted line.

Regardless of the ITI (3 min and 24 h) in the Barnes maze test, no statistically significant differences were obtained for the number of errors for the different age categories (*p* > 0.05). However, only a proportion of middle-aged and old mouse lemurs expressed more than 6 errors at 3 min ITI (4 among 15 middle-aged and 5 among 12 old animals; Figure 5A) and 24-h ITI (6 among 14 middle-aged and 5 among 12 old animals; Figure 5B). In addition, a linear regression showed a relationship between the number of errors and the age at 24 h ITI (*r*^2^ = 0.09, *p* = 0.037, Figure 5B). Whereas performances between 3 min ITI and 24 h ITI were significantly correlated (*r*^2^ = 0.18, *p* = 0.03, Table 2), a series of Spearman’s correlations did not reveal significant difference between performance on the spatial reference and working memory tasks for any age group (*p* > 0.5, Table 2). Paired test comparison between performance at 3-min and 24-h revealed that the number of errors increased significantly at 24-h delay in comparison to 3-min delay (*p* = 0.033), showing that memory declined with increasing retention intervals.

### Novel object recognition (NOR) task

**Figure 6:**
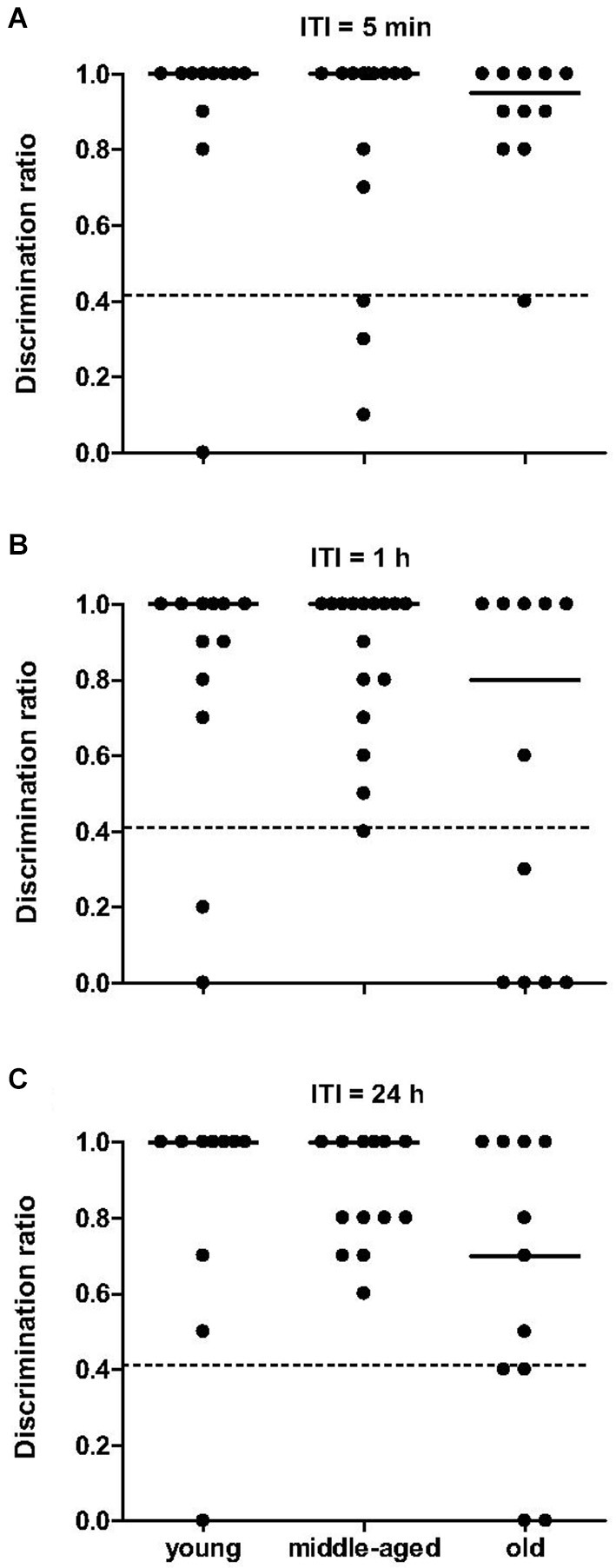
**Novel object recognition memory as function of age categories at 5 min, 1 h and 24 h**. At 5-min **(A)**, 1-h **(B)** and 24-h **(C)** retention delay, discrimination ratio did not differ across age, but identified some deficient animals (below the dotted line at 0.4). Medians are indicated by solid line.

The total time spent exploring objects did not differ between age categories, during both sample trials (*χ*^2^ = 4.6, *p* = 0.099) and choice trials (*χ*^2^ = 0.3, *p* = 0.88). Independent of the ITI, mouse lemurs of all ages spent more time exploring the novel than the familiar object (*p* < 0.05 each, Table 1). All data show that the mouse lemurs expressed object recognition memory at short- and long-term. Discrimination ratios did not differ between age categories at 5 min ITI (*χ*^2^ = 0.5, *p* = 0.77) and at 1 h ITI (*χ*^2^ = 1.4, *p* = 0.49; Figure 6). However, old mouse lemurs strongly tend to express a low discrimination ratio at 24 h ITI, in comparison to younger animals (*χ*^2^ = 5.7, *p* = 0.057; Figure 6). Interestingly, 3 out of 13 young, 3 out of 16 middle-aged and 6 out of 12 old animals had a bad discrimination ratio (less than 0.4) at 5 min ITI (Figure 6A) and/or 1 h ITI (Figure 6B). At 24 h ITI (Figure 6C), whereas only 1 among 27 young/middle-aged animals expressed a bad discrimination ratio, 4 among 11 old animals had a discrimination ratio less than 0.4. Paired test comparison between retention delays revealed no clear decrease of the discrimination ratio across time (*p* > 0.5).

### Multiparameter analysis of mnesic functions

**Figure 7:**
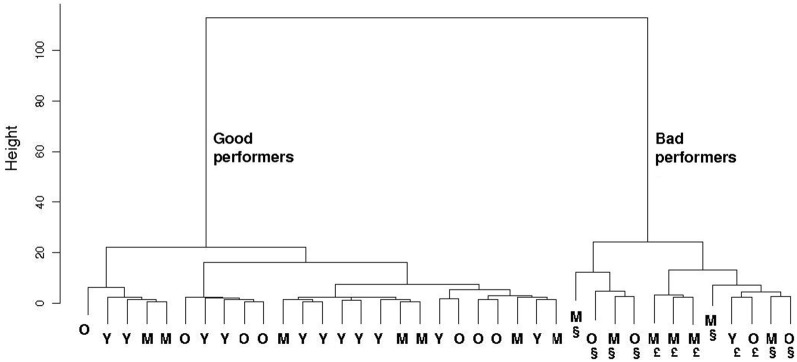
**Hierarchical clustering of both spatial reference and recognition memory parameters in the 37 evaluated mouse lemurs**. Only 1 young (Y), 7 middle-aged (M) and 4 old (O) mouse lemurs are “bad performers”. § indicates animals with memory deficit at short- and long-term. £ indicates animals with memory deficit at long-term but not at short-term.

Hierarchical cluster analysis on both spatial reference and object recognition memory tests clearly highlighted that these mnesic capacities divide the animals in two groups (Figure 7): one group with 11 young, 7 middle-aged and 7 old animals, and one other group with 1 young (49 m.o.), 7 middle-aged (66, 66, 66, 73, 80, 81 m.o.) and 4 old animals (89, 107, 109, 113 m.o.). The first group, “good performers”, is composed of 25 animals which made 2 (IQ: 1–3) errors at 3 min ITI and 3 (IQ: 2–4) errors at 24 h ITI in Barnes maze test, and expressed a discrimination ratio equal to 1 (IQ: 0.9–1) at 5 min, 1 (IQ: 0.8–1) at 1 h and 1 (IQ: 0.8–1) at 24 h in NOR task. The 12 animals of the second group, “bad performers”, made 7.5 (IQ: 5–10) errors at 3 min ITI and 11.5 (IQ: 10–14) errors at 24 h ITI in Barnes maze test, and had a discrimination ratio of 1 (IQ: 0.7–1) at 5 min, 0.8 (IQ: 0.3–1) at 1 h and 0.8 (IQ: 0.6–1) at 24 h in NOR task.

This finding suggests that only some mouse lemurs older than 49 months expressed declarative-like memory deficit, whereas majority of the other ones expressed similar cognitive capacities as younger mouse lemurs. No differences were observed between gender: 15 females and 10 males were good performers, 6 females and 6 males were bad performers.

Separated cluster analyses of mnesic performance at short- and at long-term showed that: (i) only 7 animals (4 middle-aged and 3 old; part of the second group described above) expressed short-term memory deficit, (ii) 12 animals (the same animals identified above in the second group) were impaired in long-term memory. All animals with a short-term memory deficit expressed also long-term memory impairment; however 5 animals (1 young, 3 middle-aged and 1 old; Figure 7) showed a long-term mnesic deficit but had no short-term impairment.

## Discussion

The present study was undertaken to characterize psychomotor and mnesic abilities across aging in the mouse lemur, and to identify animals showing impairments of certain mnesic functions by using a statistical method to subdivide the population into clusters according to their mnesic performance. The majority of older animals expressed psychomotor deficit and a higher exploratory activity in a novel environment. However mnesic functions did not show the same trend. Spatial working memory seems to decline progressively with age, whereas spatial reference and object recognition memories are only affected in a subset of middle-aged and old mouse lemurs.

### Independent cognitive and non-cognitive deficits

The age-related changes in psychomotor function and in exploratory behaviors may influence the mnesic performances. Indeed, impaired mnesic performances in older animals could reflect an effect of age on sensorimotor capabilities, on emotional and/or motivational status and/or on search for strategies used.

Firstly, the visual and/or psychomotor deficits could explain the poorer performances on mnesic tasks in old animals. The ophtalmological analysis revealed no evident sign of eye anomalies in any of the mouse lemurs tested, and middle-aged and old mouse lemurs expressed an intact visual discrimination performance in NOR task at 5-min and/or 1-h delays. Moreover, the analyses of the inter-relationships between the different age-sensitive behavioral parameters indicated that performances of old animals in cognitive and psychomotor tests are not significantly correlated, thus strongly suggesting that age-related decline of psychomotor and mnesic functions occurs independently. Consistent with rodent (Altun et al., 2007) and human (Woollacott and Tang, 1997) studies, psychomotor function in mouse lemur seems to be impaired in late adulthood. When compared to their young and middle-aged counterparts, old mouse lemurs showed balance and coordination deficits, and presented impaired muscle strength. Further central investigations will be designed to identify the neurobiological correlates of age-related psychomotor deficits in mouse lemurs.

Old mouse lemurs were significantly impaired in all psychomotor tests with the exception of the rotarod test. The lack of a statistically significant decrease in the rotarod performance may be due to the few animals that actually performed this test; one half of the young and one third of the middle-aged animals did not comply. Yet poorer performance in rotarod task was exhibited only in some middle-aged and old animals, suggesting that aging affected endurance of each mouse lemur in a different way. This variability in endurance, as in other psychomotor tests, could be explained by a difference in life-long physical activity that acts on muscle phenotype (Ferreira et al., 2012). Nevertheless, psychomotor impairments reach different degrees in these animals. In human, severity of deficits in psychomotor functions can differentiate healthy elderly from patients with mild cognitive impairment and patients with Alzheimer’s disease (Kluger et al., 1997; Rabbitt et al., 2006). In mouse lemurs, we found that age-related deficits in memory are independent of the psychomotor deficits that develop with age, as it has been observed in rodents (Gage et al., 1989; Miyagawa et al., 1998) and in rhesus monkeys (Lacreuse et al., 2006).

Secondly, since animals experienced novel environments and objects in memory tasks, low motivation for exploration and/or high anxiety could affect behavioral performance (Delacour and Santacana, 1967; Roozendaal et al., 2009). The open-field and light/dark plus-maze tests revealed that exploratory activity in an illuminated, novel environment increased with aging, consistent with a previous study (Némoz-Bertholet and Aujard, 2003). This could be explained by a decrease of anxiety with age, as observed in human studies (Blanchard-Fields, 2009; Larcom and Isaacowitz, 2009). Assuming that older animals seem to be less anxious than young ones, less sensitivity to mnesic impairment could be expected in old mouse lemurs. On the contrary, we observed that older mouse lemurs expressed more mnesic deficits than younger counterparts. Moreover, there was no obvious correlation between anxiety-related behaviors and the mnesic performances for each task. Altogether, the hypothesis of performance deficits due to anxiety-related behaviors can be ruled out for this species.

Age-related impairments in a given memory task were not necessarily observed in all memory tasks. This reinforces the fact that mnesic deficits in aged mouse lemur cannot be easily explained by non-cognitive decline.

### Differential age-related mnesic deficits

Spatial working memory deficit has been previously observed in some aged mouse lemurs (6–12 years) by using trained tasks: delayed discrimination (Picq, 1995) and three-panel runway tasks (Trouche et al., 2010). For the first time in mouse lemurs, we demonstrated a linear regression of the spatial working memory performance with age, via spontaneous alternation test. Spontaneous alternation performance is based on spatial working memory, but it could be explained by differences in search strategies independent of memory, such as always choosing adjacent arms. However, analyses of angle shift (number of arms visited with a different angle than the previous one, normalized to the total number of visited-2) in cross-maze revealed that few animals used a specific navigation strategy, and no differences were observed across age categories; indicating that mouse lemurs alternate spatial locations. Thus, like in humans (Davis et al., 1990; Gazzaley et al., 2005), decline in working memory seems to begin in old mouse lemur.

Whereas spatial working memory tasks imply continuous subsisting of a recent information (last visited arms in the spontaneous alternation test), spatial reference memory tasks require a short-/long-term storage of a specific information (location of the reinforcement in the Barnes maze test). Dissociation between both spatial memory processes is confirmed by the lack of correlation between the respective performances. In the Barnes maze task, the number of errors tended to increase with age at 3 min ITI (short-term memory), which became significant at 24 h ITI (long-term memory). These data demonstrate that spatial reference memory was impaired with age, which is consistent with the study of Picq et al. (2012) showing that the group of aged mouse lemurs (6.2–8.4 years) expressed significantly more errors than a young group (2.0–2.8 years). Age-related spatial reference memory decline has been equally well-documented in humans, non-human primates and rodents (Iachini et al., 2009; Sharma et al., 2010; Klencklen et al., 2012).

For the first time, NOR memory task has been investigated in mouse lemurs. Our data show that, at all ages, mouse lemurs explored more the novel objects than the familiar ones, at least when using the 5 min ITI. These findings suggest that short-term recognition memory was not altered during aging in mouse lemur, which is in agreement with rodent studies (Willig et al., 1987). With increasing delay retention of NOR task, a subset of the middle-aged and old mouse lemurs performed outside the range of the discrimination index of their young counterparts. The present results are in agreement with studies in other animal models (e.g., Rapp and Amaral, 1991; Luparini et al., 2000) and humans (e.g., Berteau-Pavy et al., 2007) that also show age-related decline in NOR memory with aging.

Although there was no within-subject correlation of performance for both spatial reference and NOR tasks, some similarities were detected between these medial temporal lobe-dependent declarative-like memory tasks. Young mouse lemurs were able to express spatial reference (Barnes maze task) and object recognition (NOR task) memories even at long delay (24 h), whereas middle-aged and old mouse lemurs performed more poorly at short- and even more at long-term. Analysis of the performances within the groups of middle-aged and old animals showed a considerable individual variability in both tasks: some performed as well as younger animals, whereas others were severely impaired. Increase of the individual variability with cognitive aging has been previously observed in this species (Picq et al., 2012), as well as in humans (Albert, 1993), monkeys (Rapp and Amaral, 1992), dogs (Salvin et al., 2011) and rats (reviewed in Gallagher, 1993). Distinction within the studied population between good and bad performers was investigated by multiparametric analysis to account for the degree of spatial reference and object recognition memory impairments. It should be noted that one young animal was a bad performer, suggesting an early deficit of memory for this animal. On the one hand, five of our middle-aged/old animals were severely deficient in both tasks, which suggests the presence of global brain dysfunction. On the other hand, some mouse lemurs were impaired in spatial reference memory, while others in object recognition memory. Spatial memory seems to be adversely impaired while object recognition memory was mildly affected. Although these two tasks are thought to rely on the medial temporal lobe, they challenge different memory processes subserved by different neural networks. NOR task probes recognition memory for single stimulus items (“context-free memory”) involving anterior subhippocampal structures such as perirhinal cortex, whereas the Barnes maze task involves relational memory that crucially depends on the hippocampus (reviewed in Mishkin et al., 1997; Buckley, 2005; Mayes et al., 2007; Iachini et al., 2009; Vann and Albasser, 2011). The patterns of impairments observed in mouse lemurs suggest that aging affects differentially these two subcomponents of the medial temporal lobe. For instance, age-related dysfunction of perirhinal cortex and hippocampal formation could explain the mnesic deficit in NOR and in spatial reference memory tasks, respectively. Interestingly, Picq et al. found that atrophy of the hippocampus and the entorhinal cortex is highly correlated with individual behavioral deficits in the Barnes maze in old mouse lemurs (Picq et al., 2012). Neuroimaging and neurochemical studies will highlight the potential brain structures related to spatial and non-spatial medial temporal lobe-dependent memory in this primate.

All these findings indicate that mnesic functions are not equally affected across aging. Since spatial reference and recognition memories are affected in human brain disorders such as Alzheimer’s disease (Burgess et al., 2006; Kessels et al., 2011), our findings may significantly expand the applicability of the mouse lemur in studying the neurobiological determinants of the cognitive deficits in normal and pathological aging. Blood and brain analyses of these phenotyped animals will complete the neurochemical and neuropathological characterization of this non-human primate model as compared to human neuropathologies.

## Conclusions

Mouse lemurs exhibit age-related behavioral impairments: psychomotor deficit starting in the late adulthood, working memory decline beginning in the old mouse lemur, while spatial reference and object recognition memory deficits are apparent only in a subgroup of middle-aged and old mouse lemurs. This pattern of cognitive aging is strikingly reminiscent of that described in humans with prefrontal lobe-dependent executive functions undergoing continuous life-long decline in normal aging whereas declarative memory undergoes only mild decline during normal aging, but is profoundly impaired in Alzheimer’s disease due to medial temporal degeneration in this pathology (Gabrieli, 1996). Accordingly, this study lends support to the notion that the mouse lemur is a suitable animal model of the different forms of human cognitive aging. Our battery of behavioral tests and the multiparametric analyses provide a useful tool to test possible pharmacological and environmental manipulations that may be able to counteract age-related decline in both psychomotor and mnesic functions, and will increase the applicability of this non-human primate model for preclinical development.

## Disclosure statement

The authors declare no conflicts of interest and no financial or personal relationships with other people or organizations that could inappropriately influence the present work. Caroline Louis and Esther Schenker are employees of Servier.

## Conflict of interest statement

The authors declare that the research was conducted in the absence of any commercial or financial relationships that could be construed as a potential conflict of interest.
